# Retracted: An Improved Ant Colony Optimization Approach for Optimization of Process Planning

**DOI:** 10.1155/2016/5960614

**Published:** 2016-03-24

**Authors:** The Scientific World Journal

The article titled “An Improved Ant Colony Optimization Approach for Optimization of Process Planning” [1] has been retracted as it was found to contain a substantial amount of material from the following published article: “A Graph-Based Ant Colony Optimization Approach for Process Planning,” by JinFeng Wang, XiaoLiang Fan, and Shuting Wan, in The Scientific World Journal.

## References

[1] Wang J., Fan X., Ding H. (2014). An improved ant colony optimization approach for optimization of process planning. *The Scientific World Journal*.

